# Evaluating the implementation of PROMs and PREMs in routine clinical care: co-design of tools from the perspective of patients and healthcare professionals

**DOI:** 10.1186/s12955-025-02333-7

**Published:** 2025-02-17

**Authors:** Clara Amat-Fernandez, Yolanda Pardo, Montse Ferrer, Guillermo Bosch, Catalina Lizano-Barrantes, Renata Briseño-Diaz, Maria Vernet-Tomas, Lluís Fumadó, Marc Beisani, Dolores Redondo-Pachón, Anna Bach-Pascual, Olatz Garin, Hospital de Mar Patient-Reported Measures Group, Clara Amat-Fernandez, Yolanda Pardo, Montse Ferrer, Guillermo Bosch, Catalina Lizano-Barrantes, Renata Briseño-Diaz, Maria Vernet-Tomas, Lluís Fumadó, Marc Beisani, Dolores Redondo-Pachón, Olatz Garin, Ángels Pont, Víctor Zamora, Nuria Argudo, Anna Bach, David Benaiges, Xavier Castells, Lluis Cecchini, Betty Chamoun, Marta Hurtado, Marta Hurtado, Alicia Noguera, Maribel Pérez-Piñero, María José Pérez-Sáez, Sonia Servitja, Cristina Siles, Montserrat Villatoro, Betty Chamoun, Jose Maria Valderas

**Affiliations:** 1 Universitat Pompeu Fabra, Barcelona, Spain; 2 Health Services Research Group, Hospital del Mar Research Institute, Doctor Aiguader 88, Barcelona, 08003 Spain; 3 CIBER en Epidemiología y Salud Pública, CIBERESP, Madrid, Spain; 4 Universitat Autònoma de Barcelona (UAB), Barcelona, Bellaterra Spain; 5 Teaching Unit of Preventive Medicine and Public Health, Hospital del Mar-ASPB-UPF, Barcelona, Spain; 6 Department of Pharmaceutical Care and Clinical Pharmacy, Faculty of Pharmacy, Universidad de Costa Rica, San José, Costa Rica; 7 Breast Diseases Unit, Hospital del Mar, Barcelona, Spain; 8 Urology Department, Hospital del Mar, Barcelona, Spain; 9 Gastrointestinal and Bariatric Surgery Unit, Hospital del Mar, Barcelona, Spain; 10 Nephrology Department, Hospital del Mar, Barcelona, Spain

**Keywords:** Implementation science, Health services research, Patient-reported outcome measures, Patient-reported experience measures, Patient centered care, Health care evaluation mechanisms, Healthcare quality assessment, Co-design, Routine clinical care

## Abstract

**Background:**

Implementation of patient-reported measures (PRMs) is an integral element for patient-centered models; however, there is still hardly any quantitative evidence regarding its impact in routine care settings. The objective of this study was to codesign two concise tools that allow for a standardized and longitudinal assessment of the implementation of PRMs in routine care in terms of acceptability and perceived value from the perspective of both patients and healthcare professionals.

**Methods:**

A list of constructs and items to be presented, separately, to patients and healthcare professionals was created from evidence gathered through a narrative literature review. Focus groups, composed of either patients or healthcare professionals from different chronic conditions, were conducted for the co-design of independent assessments. Once agreement was reached, the content validity was examined in separate consensus meetings.

**Results:**

A total of 10 patients and 10 healthcare professionals participated in the focus groups. After 7 focus groups, the PRMs Implementation Assessment Tool for patients (PRMIAT-P) was developed with 33 items in 9 constructs, and the tool for healthcare professionals (PRMIAT-HP) had 33 items in 16 constructs. Content validity was confirmed for both tools.

**Conclusions:**

The perspective of patients and healthcare professionals regarding the implementation of PRMs in routine care can be evaluated quantitively with the PRMIAT tools. These tools are understandable, concise and comprehensive, and can be used in multiple settings and for different chronic conditions. They have been codesigned as a standard set to facilitate both longitudinal assessments and performing benchmarking among different initiatives.

**Supplementary Information:**

The online version contains supplementary material available at 10.1186/s12955-025-02333-7. Permission from corresponding authors (ogarin@researchmar.net or ypardo@researchmar.net) is needed for using, sharing, or adapting these tools.

## Background

International organizations increasingly advocate for the shift toward patient-centered care models [1, 2], with the use of patient-reported measures (PRMs), both health outcomes (PROMs) [3] and experiences (PREMs) [4], being crucial to integrate patients’ voices into health services [1, 5, 6]. The systematic use of PROMs in clinical settings has been shown to improve survival [7], symptom control [8, 9], detection of unrecognized problems [10, 11], rates of emergency room visits and hospitalizations [12], communication between patients and healthcare professionals, shared decision making, and patient satisfaction [11]. On the other hand, PREMs are performance indicators that aim to collect objective and reliable information about experience in clinical encounters, allowing for quality improvement actions [13–15], and facilitating the standardization of data for long-term monitoring and benchmarking [16].

Owing to the numerous stated benefits, implementations of PROMs are underway at different levels, from micro (individual patient management) to macro (regional programs) [17, 18]. Examples of large implementations include Australia’s New South Wales region [17], Alberta’s cancer care program (Canada) [19, 20], and the UK’s National Health Service (NHS) PROMs program [21, 22]. PREMs have been implemented since 1995 through the US Consumer Assessment of Healthcare Providers and Services (CAHPS) across the Medicaid and Medicare programs [23–25]. The successful adoption of PROMs and PREMs in routine clinical care can change the organization and delivery of healthcare services. However, some requirements at different levels need to be considered, such as time, cost, response rates, or appropriate outputs; as well as an evaluation that takes into account patients’ and healthcare professionals’ needs, minimizing burdens, and understanding barriers [18].

There are several initiatives to guide healthcare systems to select and implement PROMs or PREMs in different settings [1, 23, 26–29] including some of them focused on routine clinical care [23, 29]. A systematic review has synthesized evidence on the perceived benefits and limitations of using PROMs in clinical practice as perceived by patients and healthcare professionals from qualitative studies [30].

Nevertheless, there is scarce quantitative evidence regarding the impact that PRMs implementations in routine clinical care has on patients and healthcare professionals. The ISOQOL Clinical Practice Implementation Science Work Group recommended indicators, such as the response rate of PRMs, or the percentage of healthcare professionals that attend training activities, for evaluating PRMs implementations [31], which are based on the main Implementation Science constructs (acceptability, adoption, fidelity, appropriateness, sustainability or feasibility). In addition to ad-hoc qualitative studies [30, 32], and the aforementioned proposal on potential indicators, a structured quantitative method has been used in some studies to evaluate the degree to which the routine implementation of PRMs has been successful [33, 34]. Recently, a study aimed at identifying barriers and facilitators postimplementation from the perspectives of healthcare professionals and patients, with a codesigned an ad-hoc survey, revealed the necessity of ongoing efforts to ensure a successful hospital-wide PROM implementation [35]. Although, these studies did quantitively measure the impact of their implementations, the tools employed were only used for PROMs implementation, they were not designed for longitudinal use and their development was not properly described. Moreover, a narrative review of the use of PREMs highlights, not only the scarcity of longitudinal information from PREM initiatives, but also the lack of evidence of its impact [15].

To the best of our knowledge, standardized tools that measure the impact of both PROMs and PREMs implementations in routine clinical care from the perspective of patients and healthcare professionals do not exist. Thus, the objective of this study was to codesign concise tools that allow for a standardized and longitudinal assessment of the implementation of PRMs in routine clinical care, from the patients’ and healthcare professionals’ perspectives independently, in terms of acceptability and perceived value. The tools aim to be applied at different institutions and regions, to evaluate the implementation of PRMs regardless of the setting or condition.

## Methods

In the absence of specific procedures, international guidelines for PROMs development [36–38] were followed. First, to identify relevant areas in which PRMs implementation may impact patients and healthcare professionals, a narrative literature review [39] on the assessment of PRMs implementations was conducted. Second, the identified areas were used to initiate a codesign process that included focus groups with patients and healthcare professionals to define, select, and properly formulate the content of the tools for PRMs implementation assessment. Content validity was examined through consensus meetings with patients and PRMs experts.

The study adhered to the Consolidated Criteria for Reporting Qualitative Research (COREQ) checklist [40], a tool for reporting qualitative studies.

### Literature review

A narrative literature review of published evidence was conducted to identify existing evaluation strategies for PRMs implementation initiatives through either qualitative or quantitative methodologies. This search was performed in PubMed in November 2022, without restrictions on the publication dates, using terms such as: quality of life, PROM, PREM, implementation, routine use, evaluation, assessment, and impact. The backward and forward citation tracking method was also used to capture literature that did not appear directly in the search. The inclusion criteria were as follows: description of a PRM implementation via an Implementation Science framework, evaluation of a PRM implementation through surveys or qualitative methodologies, exploration of barriers and facilitators of a PRM implementation in routine practice, and implementations of PRMs at any level of the health system or in any pathology. The exclusion criteria were implementations of PRMs without an evaluation being described and implementation initiatives that were not specific for routine clinical practice.

### Codesign of the assessment tools

The relevant areas identified in the literature review were extracted and edited into a content list to be presented in the focus groups, as the next step of the development process [38]. Focus groups took place at the hospital’s research institute and started with a brief explanation of the PROMs, PREMs and project objectives, and were moderated by three of the researchers following a semi structured format [37].

The participants were patients in active management for breast or prostate cancer, chronic kidney disease, or bariatric surgery purposively selected, and health professionals from hospital departments participating in existing research studies with PROMs. All participants gave prior written informed consent. The focus groups were stratified by condition and conducted separately for patients and healthcare professionals. Patients’ age, gender, basic clinical variables and housing area codes were collected from their medical records. Healthcare professionals’ age, gender, job/role, years of experience, and years working in the institution were also collected.

Phenomenological inquiry was used to understand the participants’ perspectives and detect new constructs or items, modify or remove those already detected in the literature, and assess their relevance and importance through discussion until agreement and saturation were reached [41]. The researchers made the final decision at the end of each focus group if no agreement among the participants was reached. The response options were also discussed in each group. This process was replicated for all focus groups to ensure that the content represented a wide range of health conditions. An updated version was produced by the research team after each focus group and presented to the following group. The items of both tools (patients and healthcare professionals’ versions) were developed simultaneously in Catalan and Spanish during the focus groups. All focus groups were audio recorded and transcribed with the software HappyScribe Ltd.© [42] to ensure that the distinct versions of the item lists gathered all the updates agreed upon by the participants. Researchers took notes during the focus groups to guide the changes to be made to the content list.

### Content validity

Content validity was examined through two consensus meetings that were organized to judge the clarity, comprehensiveness, relevance, and redundancy of each item of the tools [43, 44]. In the case of the patients’ tool, one participant from each condition was selected to participate, all of whom were expert patients. For the healthcare professionals’ tool, a consensus meeting was organized with the PRMs experts, none of whom had participated in the focus groups. The guide developed beforehand for each meeting included 4 questions regarding comprehensibility, adequacy, and importance, together with a final question about whether participants would change anything in the item. The resulting tools were sent to the participants of the focus groups for their feedback.

### Ethics

This study was approved by the Ethics Committee of Parc de Salut Mar (Barcelona, Spain).

## Results

Among the articles produced by the search in PubMed, 50 were considered relevant for the present study. From those finally included in the narrative review of the literature, several domains were selected that focused on patients’ and healthcare professionals’ perspectives (7 from the Consolidated Framework for Implementation Research [45], and 8 from the Proctor framework [46]): patients’ needs and resources [45], structural characteristics [45], sustainability [46], knowledge and beliefs about the intervention [45, 46], adoption [46], self-efficacy [45], and individual state of change [45]. Moreover, after also reviewing the qualitative evidence from the literature, 15 items covering 7 different constructs were presented to patients in the focus groups; and 24 items, covering 14 constructs, were presented to healthcare professionals [30, 32, 45–56]. These areas along with a study on the application of Implementation Science theories to inform the use of PROMs in routine clinical care, guided the initial content list for the focus groups [48].

In total, 10 patients and 10 healthcare professionals participated in the focus groups (the participants’ demographic information is shown in Table 1). A total of 50% of the patients were female, aged 40–77 years, and had diverse socioeconomic backgrounds [57]. The average time since diagnosis of their main pathology was 9 years (range 1–42 years), and most of the participants (8/10) had undergone at least one surgery. Most of the healthcare professionals who participated were female (7/10), and half of them were < 50 years old. On average, the healthcare professionals had 19.2 years of experience in their profession (ranging from 8–37 years). The main reason for not participating was time availability by both patients and healthcare professionals.

**Table 1:** Participant characteristics

**PATIENTS**	All (*n* = 10)	Breast cancer (*n* = 2)	Chronic kidney disease (*n* = 2)	Bariatric surgery (*n* = 3)	Prostate cancer (*n* = 3)
**Gender, n**					
Male	5	-	1	1	3
Female	5	2	1	2	-
**Age, n**					
40–49 years	2	-	-	2	-
50–59 years	3	1	1	1	-
60–69 years	4	1	1	-	2
70–79 years	1	-	-	-	1
Socioeconomic index, n*					
High	3	-	-	1	2
Middle	3	2	-	1	-
Low	3	-	2	-	1
Very low	1	-	-	1	-
**Time since diagnostic in years, mean (min–max)**	9.0 (1–42)	10.0 (3.5–16.5)	21.5 (1–42)	4.8 (1.5–9)	4.0 (1–8)
**Time since last treatment / intervention in years, mean (min–max)**	3.3 (0–10.5)	6.8 (3–10.5)	2.0 (0–4)	2.2 (0–5)	3.0 (0.5–5.5)
**HEALTHCARE PROFESSIONALS**	All (*n* = 10)	Breast cancer (*n* = 4)	Chronic kidney disease (*n* = 2)	Bariatric surgery (*n* = 3)	Prostate cancer (*n *= 1)
**Gender, n**					
Male	3	-	-	2	1
Female	7	4	2	1	-
**Age, n**					
30–39 years	2	1	1	-	-
40–49 years	5	1	1	2	1
50–59 years	3	2	-	1	-
**Health Professional, n**					
Doctor	7	3	1	2	1
Nurse / Case manager	2	1	1	-	-
Nutritionist	1	-	-	1	-
**Years of experience, mean (min–max)**	19.2 (8–37)	25.3 (9–37)	14.7 (13–17)	14.0 (8–20)	19.0
**Years in the current institution, mean (min–max)**	16.5 (1–31)	21.8 (14–31)	13.1 (10–17)	12.3 (1–18)	14.0

*Socioeconomic status was estimated from the area code via a deprivation index developed to strengthen equity among territories in the allocation of resources for primary care services [57]

**Fig. 1:**
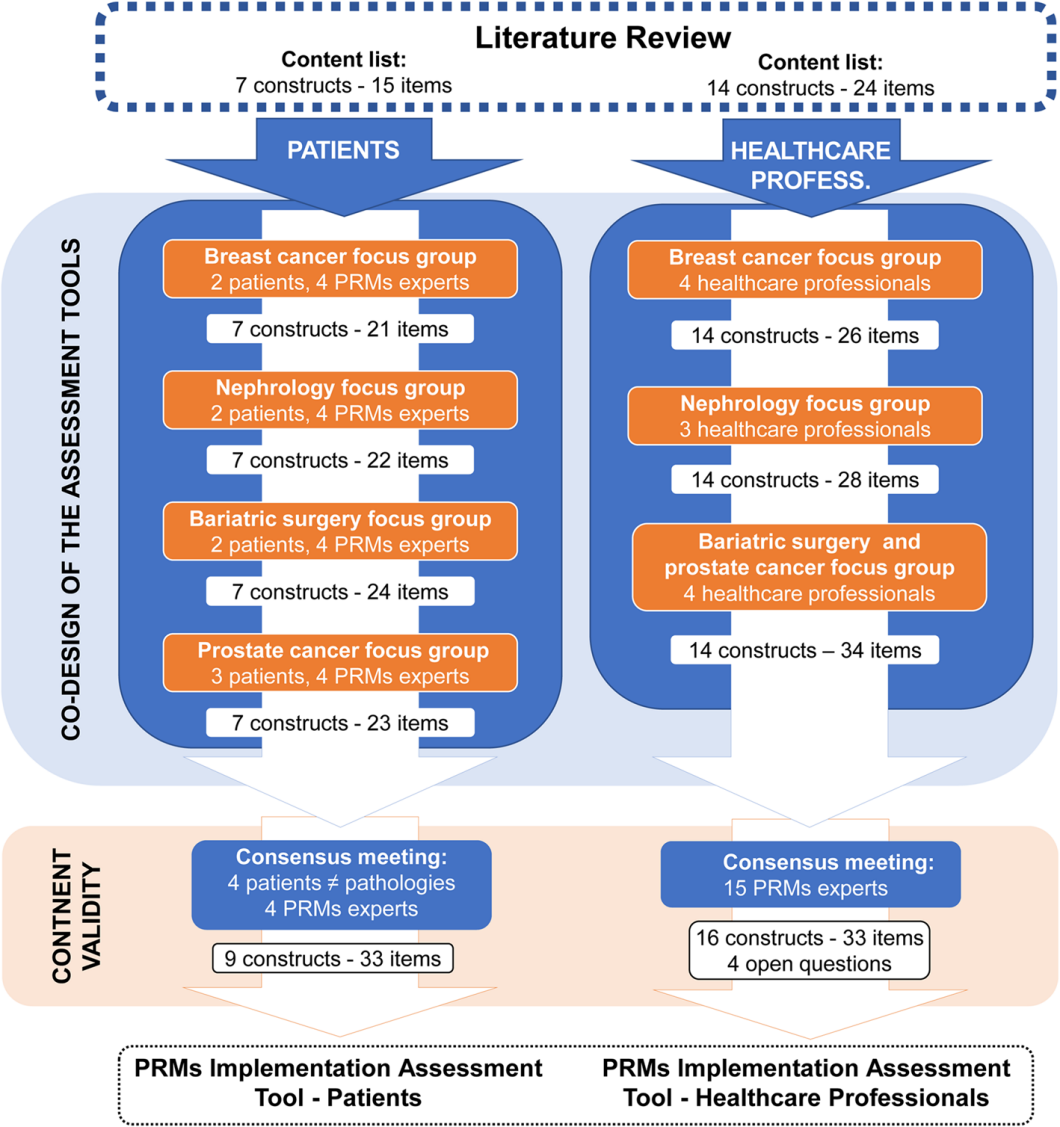
Development process of the PRMs Implementation Assessment Tools

Figure 1 shows the steps followed for the development of the two PRM Implementation Assessment Tools (PRMIAT) and the number of constructs and items at the end of each of these steps. After the 4 focus groups with patients, the instrument for patients contained 23 items in 7 constructs. After the 3 focus groups with healthcare professionals, the instrument for healthcare professionals contained 34 items in 14 constructs. Saturation was reached in the last focus groups, as the same themes had come out repeatedly previously. The focus groups lasted between 80 and 120 min.

The content validity evaluation of the patients’ tool was completed in a 60-min consensus meeting with 8 participants (one patient with each condition and 4 investigators). Six items were reformulated for better comprehension. Alterations included editing terminology such as changing “healthcare center” to “hospital”, incorporating examples within the items, modifying the verbal tense, changing the direction of the item, and shortening the items. It was ensured that the wording remained adequate for patients with different conditions; items were aligned with the concepts they were intended to measure and maintained relevancy. Redundancy and completeness were evaluated, resulting in the addition of 6 items related to the constructs of ‘suitability for all patients’, and ‘value of the implementation’. Separate sections were designated within the tool to accommodate the different aspects of PROMs and PREMs independently. Additionally, four items addressing usability were included.

The content validity of the healthcare professionals’ tool was appraised in a 90-min consensus meeting of 15 PRMs experts (epidemiologists, public health researchers, psychometricians, and medical residents). As a result, two items were excluded due to low relevance and one new item was added from splitting an item into two, focusing on PROMs’ and PREMs’ implementation separately. Five items were edited for better comprehensibility by adjusting the verbal tense or restructuring the question, whereas seven items required substantial revisions to clarify ambiguous language or provide additional detail. Four open-ended questions were appended to elicit insights into the advantages, disadvantages, barriers encountered, and suggestions to improve the usability of the platform being used to complete the PRMs.

**Fig. 2:**
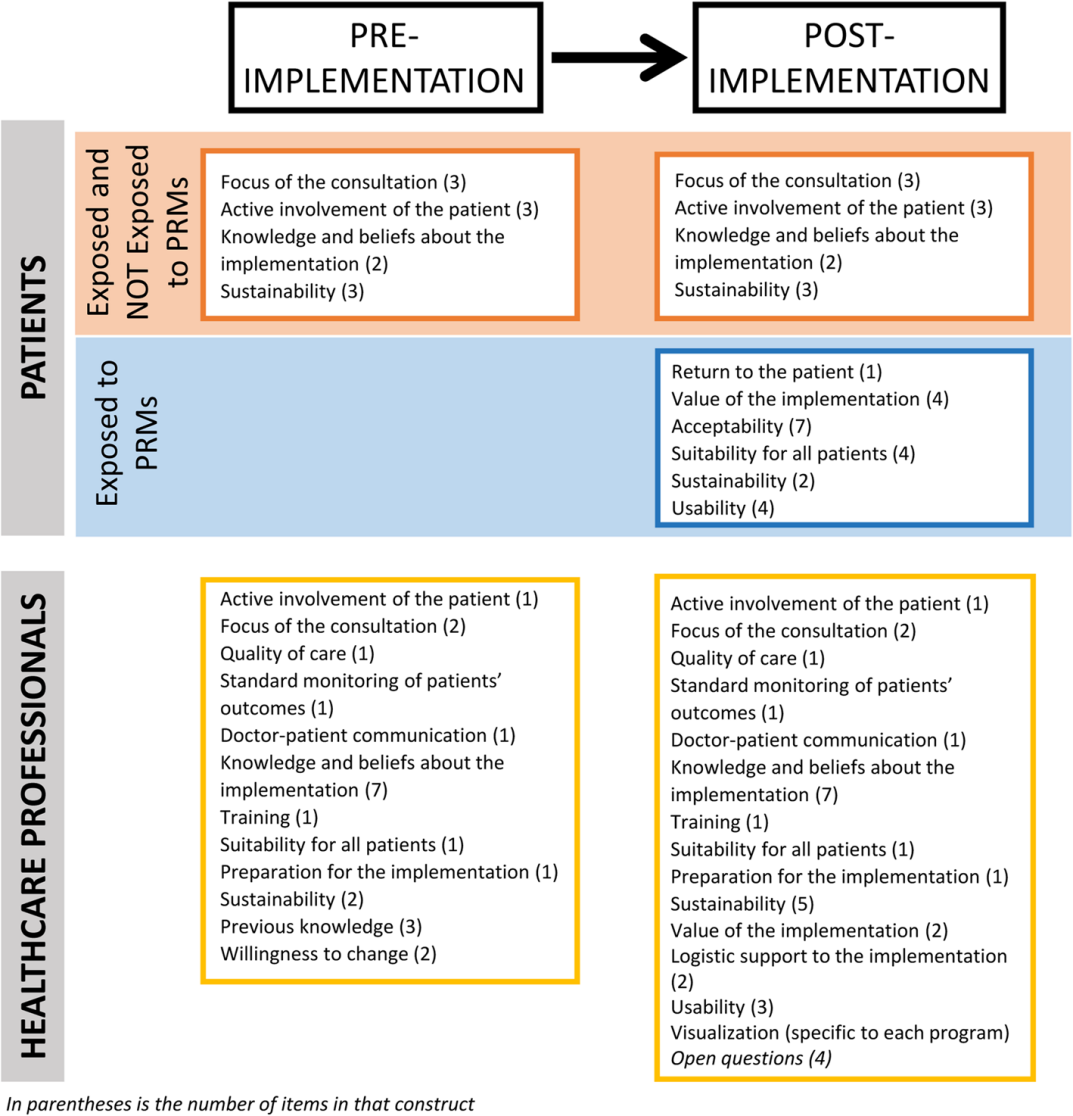
Constructs and administration timeline for the PRMs Implementation Assessment Tools for patients and healthcare professionals

The final PRM Implementation Assessment Tool – Patients (PRMIAT-P) is composed of 33 items that cover 9 constructs (Table 2). It is divided into 4 sections: 1) to be answered independently by all patients regardless of previous exposure to PRMs as part of their routine clinical care (11 items), 2) on the implementation of PROMs (13 items), 3) on the implementation of PREMs (5 items), and 4) usability questions (4 items). Sections 2–4 have been developed to be administered after patients have answered PROMs, PREMS, or both (Fig. 2).

**Table 2:** Constructs and items of the PRMs Implementation Assessment Tools for patients

Constructs	Items
**Focus of the consultation**^**a**^	• Understanding of health status • Talked about important topics of daily life • Doctor has overall view of health status
**Active involvement of the patient**^**a**^	• Visit preparation • Participation at the hospital has increased recently • The image of the hospital has improved recently
**Knowledge and beliefs about the implementation**^**a**^	• Answering health questionnaires improves the care received • It is useful to evaluate the care received
**Return to the patient**^**b**^	• Professionals discuss responses to health questionnaire with patient
**Value of the implementation**^**b**^	• Questionnaires help communicate with healthcare professionals • Questionnaires help remember to share symptoms to healthcare professionals • Help sharing uncomfortable issues • Facilitate that psychological issues be discussed and addressed
**Acceptability**^**b,c**^	• Frequency to answer health questionnaires is adequate • Frequency to answer experience questionnaires is adequate • Time is takes to respond health questionnaires is adequate • Time is takes to respond experience questionnaires is adequate • Health-related questions are repetitive • Experience-related questions are repetitive • Questions are adequate for current health status
**Suitability for all patients**^**b,c**^	• Questionnaires include all relevant information • Health questionnaires are reassuring • Health questionnaires create anxiety
**Usability**^**b,c**^	• Program used to answer questionnaires is easy to use • Display of the questions is good • Wording of the questions is understandable • Answering questions electronically facilitates participation
**Sustainability**^**a,b,c**^	• Willing to answer health questionnaires • Willing to answer questions about the care received • Willing to answer questionnaires on personal electronic devices • Would like to continue answering questionnaires

^a^General to PRMs implementations

^b^PROMs implementation

^c^PREMs implementation

The PRM Implementation Assessment Tool – Healthcare Professionals’ (PRMIAT-HP) is composed of 33 items representing 16 constructs (Table 3). The tool closes with 4 open questions to be answered once the PRMs have been implemented. The open questions were added to identify the advantages and disadvantages of the implementation that have yet to be detected or may be specific to a department or service, the usability issues they encountered, and integration problems with the current visualization software. It is divided into time sections: before the implementation program has begun (24 items), once PRMs have been implemented, or at both time points (29 items) (Fig. 2).

**Table 3:** Constructs and items of the PRMs Implementation Assessment Tools for healthcare professionals

Constructs	Items
**PRE-IMPLEMENTATION SECTION**	
** Previous knowledge**	• Understanding of what PROMs are • Interpreting PROMs results • Understanding of what PREMs are
** Willingness to change**	• Willingness to implement PROMs in clinical practice • Reason for not willing
**PRE AND POST IMPLEMENTATION SECTION**	
** Active involvement of the patient**	• Patients actively involved in disease management
** Focus of the consultation**	• Doctor has overall view of health status of patients • Identifying main needs of patients
** Quality of care**	• Satisfaction with the quality of care towards patients
** Standard monitoring of patients’ outcomes**	• Standardized visits for patients at the same stage of the disease
** Doctor-patient communication**	• Communication with patients is fluid
** Suitability for all patients**	• The PROMs used collect all relevant information
** Knowledge and beliefs about the implementation**	• Current use of PROMs in routine practice • Implementation of PROMs provides benefits for patients • Implementation of PROMs provides benefits for professionals • Implementation of PROMs provides benefits for professionals’ institution/department/service • Implementation of PREMs provides benefits for patients • Implementation of PREMs provides benefits for professionals • Implementation of PREMs provides benefits for professionals’ institution/department/service
** Training**	• Sufficient training to interpret and use PROMs
** Preparation for the implementation**	• Institution is prepared to incorporate PROMs into clinical practice
** Sustainability**	• Use of PROMs will be incorporated into routine clinical practice • Use of PREMs will be incorporated into routine clinical practice
**POST-IMPLEMENTATION SECTION**	
** Sustainability**	• Incorporation of PROMs into own routine clinical practice • Institution should support the implementation of PRMs • Continuing to use PRMs beyond current implementation program
** Value of the implementation**	• Incorporation of PROMs has improved overall view of the patient • Detection of symptoms sooner
** Logistic support to the implementation**	• Knowing who to contact regarding questions about the implementation • Enough support from research team when needed
** Usability**	• PROMs administration’s frequency is adequate • PROMs results are easily accessible • PROMs results are easily interpretable
** Open questions**	• Advantages of PRMs implementation program • Disadvantages of PRMs implementation program • How can the usability of the software be improved • What is missing in the software used

Both tools use a 5-point Likert scale ranging from ‘Strongly disagree’ to ‘Strongly agree’ [58]. The participants confirmed the adequacy of this response scale when presented at each focus group and at the two consensus meetings. See supplementary file 1 for the full version of the questionnaire.

## Discussion

The objective of this study was to codesign concise tools with relevant stakeholders, that allow for a standardized and longitudinal assessment of the implementation of PRMs in routine clinical care in terms of acceptability and perceived value. The developed PRMIAT tools (PRMIAT-P and PRMIAT-HP) differ from previous assessment strategies [33–35, 48, 55, 59] by providing independent and different information from patients and healthcare professionals; being applicable in different settings, institutions, and regions; evaluating the impact of administering PROMs and/or PREMs; and measuring change in the stakeholders’ perception throughout their implementation.

Among the PRM implementation initiatives that have previously been quantitively evaluated, three [33, 35, 48] stand out for meeting several of the abovementioned criteria. One of them applied the Proctor implementation framework [46] to develop indicators to evaluate the implementation of PROMs in several conditions [48] from the perspective of patients and clinicians. However, these indicators were not codesigned with patients or healthcare professionals, PREMs were not included, and the evaluation of this implementation was not published. The second initiative developed the Medical Care Questionnaire (MCQ) to evaluate PROMs implementation in a randomized control trial of oncology patients [33, 60]. The MCQ measures 3 domains (communication, coordination, and patient preferences) at different timepoints, administering an end-of-study questionnaire to patients and clinicians to evaluate usability, content, relevance, usefulness, and reliability of the use of PROMs in routine care. However, since the MCQ also covers the experience of the patient with the healthcare system, it may not be suitable for evaluating an implementation that also includes PREMs in routine care [60]. The third initiative developed a survey based on interviews with patients and healthcare professionals focused on identifying enablers and barriers to be administered only after the implementation of PROMs [35].

Previous studies have identified different barriers for patients (time burden, poor usability, lack of feedback) than for healthcare professionals (lack of knowledge, integration in their workflow, difficult access and interpretation of results) [47, 49, 61–64]. In the present study, several constructs were included in both the PRMIAT-P and the PRMIAT-HP, indicating that certain areas of interest and key features of a PRM implementation, such as the focus of the consultation or knowledge and beliefs about the implementation, are shared between the patients and the healthcare professionals. However, the return of information to the patient [11], an important indicator of a successful implementation, was relevant only for patients in our study, whereas logistic support for the implementation program and training were constructs relevant only to healthcare professionals, thus were only included in their questionnaire.

The PRMIAT tools make it possible to measure patients’ and healthcare professionals’ perspectives before and after PRMs have been implemented. For example, healthcare professionals are asked about the degree to which they think that PRMs benefit their patients, which should change over time if PRMs are being used routinely. To our knowledge, there is only one ad-hoc questionnaire [34] that allows for the evaluation of healthcare professionals’ perspectives before and after the implementation of PROMs; however, it does not evaluate patients’ perspectives. The majority of PRMs implementation assessments (qualitative or quantitative) only assess patients or healthcare professionals once the implementation has begun [12, 30, 35, 51]. A major difficulty in measuring change is related to presenting patients with questions about hypothetical concepts and tools that they have yet to use and understand, which requires that some constructs be presented in the conditional tense. This difficulty could explain why previous evaluations did not measure change. However, it is particularly important to be able to measure longitudinal changes in perspective toward implementation by stakeholders before and after PRMs are integrated into routine workflows.

Because of this aspect of the tool, being able to detect changes before and after PRMS implementation, together with the fact that it can be used to assess the impact of both PROMs and/or PREMs’ use, the PRMIAT is divided into 4 sections that can be used independently according to the characteristics of the implementation program. A recently published review emphasized the lack of longitudinal and quantitative data describing the impact of the use of PREMs [15]. In fact, the PRMIAT is remarkable for enabling also the evaluation of PREMs’ implementation, in contrast to most published evidence, which focuses predominantly on PROMs [34, 35, 48, 56].

From a methodological standpoint, codesigning with the participation of different stakeholders — in this case patients, healthcare professionals and PRMs experts — is a key factor to ensure the content validity of the new instrument [34, 55, 56]. Taking healthcare professionals’ and patients’ voices into account is crucial [28, 51, 63, 65] to be aware of the concrete benefits of using PRMs routinely, not just assuming the positive results obtained from clinical trials, but also identifying enablers and barriers for all the actors involved. Nevertheless, none of the previous studies that used a survey or questionnaire to assess the impact of a PRM implementation evaluated the content validity of the tool as part of their development, nor assessed its psychometric properties once the tools had been administered.

In the present study, the COSMIN methodology for evaluating the content validity of PROMs [66] was used to drive the consensus meetings. Even though that methodology was developed to be used for systematic reviews in which the content validity of several PROMs is evaluated and compared, it can also be used as a guide when developing a structured questionnaire. In that sense, researchers reviewed the COSMIN-proposed criteria to ensure good content validity, summarizing the relevance, comprehensiveness, and comprehensibility of the PRMIAT-P and PRMIAT-HP.

Once the PRMIAT tools are administered to evaluate a PRMs implementation in routine clinical care, they should be complemented with process indicators for institutional strategic purposes depending on the level of the implementation, in addition to cost measures as recommended in previous studies [31, 48]. All the complementary indicators, including those related to the use of e-platforms to collect PRMs, should be specifically selected for each program to gain a comprehensive understanding of the impact of that specific implementation.

### Limitations

While this study provides a valuable tool for PRMs implementations, which are increasingly common worldwide, several limitations should be acknowledged. First, the tools developed in the present study could have been composed of other items if participants had belonged to other health units or if their participation order had been different. Nevertheless, the selected conditions represent a wide range of healthcare processes, and initial constructs were maintained throughout the development process; thus, all groups had the chance to discuss and complement them. Second, the researchers leading the focus groups and consensus meetings potentially had a bias toward the benefits of the PRM implementation, due to their background, that could have steered the discussions. To avoid this bias, participants were informed of the reason why this study was designed, and of the most prevalent benefits and hindrances found in the literature. Moreover, the research team did not have any relationship with the patients prior to the study; however, they did have a relationship as collaborators with the healthcare professionals who participated in the focus groups. Third, the tools presented here were developed simultaneously in Catalan and Spanish; thus, for their proper utilization outside of the Spanish healthcare context, a cultural and linguistic adaptation should be performed.

Additionally, the focus groups were all recorded, but the transcription was only used by the research team to support changes in the content of the tools derived from paper notes; they were therefore not analyzed. Lastly, each focus group with patients and healthcare professionals in each condition had a limited number of participants, though the total number of participants was in line with qualitative studies (10 patients and 10 healthcare professionals). The working team of the project includes 3–5 expert patients and 2–5 healthcare professionals per group, and all were invited to the focus groups, although not all could attend on the date agreed upon. However, in the process of recruiting and selecting patient participants, it was ensured that the patients had undergone different types of treatments, to achieve heterogeneity within each focus group. Similarly, the healthcare professionals who participated were from different disciplines within the care of the same health condition. Separating patients based on their clinical condition for the focus groups was a conscious decision by the research team, to allow the different experiences of patients and healthcare professionals to influence the development of tools.

## Conclusions

The PRMIAT tool is the first approach that allows for standardized evaluations of PROMs and PREMs implemented in routine clinical practice, considering the perspective of the main actors and beneficiaries of these programs. Both instruments, PRMIAT-P and PRMIAT-HP, are understandable, appropriate for their intended use, comprehensive, and can be easily used in multiple settings and for different chronic conditions. They have been designed to become a standard set for the assessment of the implementation of PRMs, facilitating repeated evaluations in the same center (to assure improvement), and comparisons of different implementation frameworks, approaches, and administration or visualization platforms, becoming a benchmark to compare institutions or regions. The real-world implementation of PRMs can impact patients’ empowerment, health monitoring, or health care quality. This impact needs to be confirmed through its assessment, and the PRMIAT can be the tool to achieve it.

## Supplementary Information

Supplementary Material 1.

## Data Availability

No datasets were generated or analysed during the current study.

## Abbreviations

CAPHS: Consumer assessment of healthcare providers and systems
MCQ: Medical Care Questionnaire
NHS: National Health Service
PREMs: Patient-Reported Experience Measures
PRMs: Patient-Reported Measures
PROMs: Patient-Reported Outcome Measures
PRMIAT: PRMs Implementation Assessment Tool
PRMIAT-HP: PRM Implementation Assessment Tool-Healthcare Professionals
PRMIAT-P: PRM Implementation Assessment Tool-Patients
