# Supplementary material for: Evaluating the implementation of PROMs and PREMs in routine clinical care: co-design of tools from the perspective of patients and healthcare professionals
Source: Health Qual Life Outcomes. 2025 Feb 17;23:15. doi: 10.1186/s12955-025-02333-7 (PMC11834580; doi:10.1186/s12955-025-02333-7)
Supplement: Supplementary file 1 — Supplementary Material 1. [file 12955_2025_2333_MOESM1_ESM.pdf]

Supplementary material

Title: Evaluating the Implementation of PROMs and PREMs in Routine Clinical Care: Co-design of Tools from the Perspective of Patients and Healthcare Professionals

Journal: Health and Quality of Life Outcomes

SUPPLEMENTARY INFORMATION

PRM Implementation Assessment Tool – Patients (English)

Questionnaire to evaluate the implementation of PROMs and PREMs in routine clinical practice at Hospital del Mar

Evaluation of the implementation program (for all patients)

Instructions: the following questions refer to the research project "Implementation of PROMs and PREMs in routine clinical care: evaluation of their requirements and impact" in which you participate. The answers to this questionnaire will be treated anonymously. To answer, read each question in the order they appear and choose the response option that best reflects your opinion

		Strongly disagree	Disagree	Neither agree nor disagree	Agree	Strongly agree
1	I have a good understanding of my health status.	☐	☐	☐	☐	☐
2	In the last visits, we have talked about important topics for my daily life (e.g. mobility issues, psychological problems, other illnesses, the impact of my illness on my daily activities, dietary and physical exercise guidelines, etc.).	☐	☐	☐	☐	☐
3	The doctor has a global view (e.g. emotional aspects, the effect of the illness on daily activities) of my health status related to my illness.	☐	☐	☐	☐	☐
4	I prepare for my visits before going to the hospital (e.g. by writing down questions or symptoms).	☐	☐	☐	☐	☐
5	My participation at the hospital has increased in recent months (e.g. participating in studies, patient groups, as a patient expert, giving my opinion, etc.).	☐	☐	☐	☐	☐
6	I am willing to answer <u>questionnaires about my health status</u> sent by the hospital before my next visit.	☐	☐	☐	☐	☐
7	Answering <u>questionnaires about my health status</u> sent by the hospital improves the care I receive.	☐	☐	☐	☐	☐
8	I am willing to answer questions about the <u>care received</u> at the hospital.	☐	☐	☐	☐	☐
9	I consider it useful to evaluate the <u>care received</u> at the hospital.	☐	☐	☐	☐	☐

SUPPLEMENTARY INFORMATION

10	I am willing to respond to questionnaires sent by the hospital on personal electronic devices (mobile phone, computer, tablet).	☐	☐	☐	☐	☐
11	The image I have of the hospital has improved in recent months.	☐	☐	☐	☐	☐

Evaluation of administration

	**PROMS SECTION**<br>Regarding the health questionnaires sent to my electronic device by the hospital:	**Strongly disagree**	**Disagree**	**Neither agree nor disagree**	**Agree**	**Strongly agree**
1	They include all the relevant information to me.	☐	☐	☐	☐	☐
2	The frequency at which I have to respond is adequate.	☐	☐	☐	☐	☐
3	The time it takes to respond is adequate.	☐	☐	☐	☐	☐
4	The questions are repetitive.	☐	☐	☐	☐	☐
5	The questions are adequate for my current health status.	☐	☐	☐	☐	☐
6	During the visit, the professional has discussed my responses with me.	☐	☐	☐	☐	☐
7	I would like to continue answering questionnaires about my health status.	☐	☐	☐	☐	☐
8	They help me communicate with the health professionals.	☐	☐	☐	☐	☐
9	They help me remember to share symptoms with the health professional that I may have forgotten about.	☐	☐	☐	☐	☐
10	They help me share uncomfortable issues that I may be embarrassed to communicate normally.	☐	☐	☐	☐	☐
11	They facilitate that psychological issues be discussed and addressed during the visit.	☐	☐	☐	☐	☐
12	They reassure me.	☐	☐	☐	☐	☐
13	They make me anxious.	☐	☐	☐	☐	☐

SUPPLEMENTARY INFORMATION

	**PREMS SECTION**<br>Regarding the <u>questionnaires about the care I have received</u> that the hospital sends to my electronic device:	**Strongly disagree**	**Disagree**	**Neither agree nor disagree**	**Agree**	**Strongly agree**
1	They include all the relevant information to me.	☐	☐	☐	☐	☐
2	The frequency at which I have to respond is adequate.	☐	☐	☐	☐	☐
3	The time it takes to respond is adequate.	☐	☐	☐	☐	☐
4	The questions are repetitive.	☐	☐	☐	☐	☐
5	I would like to continue answering questionnaires about the <u>care I have received</u> at the hospital.	☐	☐	☐	☐	☐

	**PRMS SECTION**<br>Regarding the <u>questionnaires</u> that I receive from the hospital:	**Strongly disagree**	**Disagree**	**Neither agree nor disagree**	**Agree**	**Strongly agree**
1	I consider the tool/program used to be easy to use.	☐	☐	☐	☐	☐
2	The display of the questions is good.	☐	☐	☐	☐	☐
3	The wording and language of the questions is understandable.	☐	☐	☐	☐	☐
4	Having to answer the questions electronically facilitates my participation.	☐	☐	☐	☐	☐
5	I consider the tool/program used to be easy to use.	☐	☐	☐	☐	☐

SUPPLEMENTARY INFORMATION

PRM Implementation Assessment Tool– Patients (Spanish)

<u>Cuestionario de evaluación de la implementación de PROMS y PREMS a la práctica clínica rutinaria en el Hospital del Mar</u>

<u>Evaluación del programa de implementación (para todos los participantes)</u>

<u>Instrucciones:</u> las siguientes preguntas hacen referencia al proyecto de investigación "Implementación de PROMs y PREMs en la atención clínica rutinaria: evaluación de sus requisitos e impacto" en el que usted participa. Las respuestas a este cuestionario serán tratadas de manera anónima. Para contestar, lea cada pregunta en el orden que aparecen y elija la opción de respuesta que mejor refleje su opinión.

		Totalmente en desacuerdo	En desacuerdo	Ni de acuerdo ni en desacuerdo	De acuerdo	Totalmente de acuerdo
1	Tengo un buen conocimiento de mi estado de salud.	☐	☐	☐	☐	☐
2	En las últimas visitas, se ha hablado sobre temas importantes para mi día a día (ej: problemas de movilidad, problemas psicológicos, otras enfermedades, el impacto de mi enfermedad en mis actividades diarias, pautas de alimentación y ejercicio físico, etc.).	☐	☐	☐	☐	☐
3	El/la doctor/a tiene una visión global (ej: aspectos emocionales, efecto de la enfermedad sobre las actividades diarias) de mi estado de salud relacionado con mi enfermedad.	☐	☐	☐	☐	☐
4	Me preparo las visitas antes de ir al hospital (ej: apuntándome preguntas o síntomas).	☐	☐	☐	☐	☐
5	Mi participación en el hospital ha aumentado en los últimos meses (ej: participando en estudios, en grupos de pacientes, como paciente experto, dando mi opinión, etc.).	☐	☐	☐	☐	☐
6	Estoy dispuesto/a a responder <u>cuestionarios sobre mi estado de salud</u> que me envíe el hospital antes de la próxima visita.	☐	☐	☐	☐	☐

SUPPLEMENTARY INFORMATION

7	Responder cuestionarios sobre mi estado de salud que me envíe el hospital mejora la atención que recibo.	☐	☐	☐	☐	☐
8	Estoy dispuesto/a a responder preguntas sobre la atención recibida en el hospital.	☐	☐	☐	☐	☐
9	Considero útil valorar la atención recibida en el hospital.	☐	☐	☐	☐	☐
10	Estoy dispuesto/a a responder en dispositivos electrónicos personales (móvil, ordenador, tablet) a cuestionarios que me envíe el hospital.	☐	☐	☐	☐	☐
11	La imagen que tengo del hospital ha mejorado en los últimos meses.	☐	☐	☐	☐	☐

Evaluación de la administración

	**BLOQUE PROMS**<br>Respecto a los cuestionarios de salud que me envía el hospital a mi dispositivo electrónico:	**Totalmente en desacuerdo**	**En desacuerdo**	**Ni de acuerdo ni en desacuerdo**	**De acuerdo**	**Totalmente de acuerdo**
1	Incluyen toda la información relevante para mí.	☐	☐	☐	☐	☐
2	La frecuencia en la que debo responder es adecuada.	☐	☐	☐	☐	☐
3	El tiempo que se tarda en responder es adecuado.	☐	☐	☐	☐	☐
4	Las preguntas son repetitivas.	☐	☐	☐	☐	☐
5	Las preguntas se adecuan a mi estado de salud actual.	☐	☐	☐	☐	☐
6	Durante la visita, el profesional ha comentado conmigo mis respuestas.	☐	☐	☐	☐	☐
7	Me gustaría seguir respondiendo cuestionarios sobre mi estado de salud.	☐	☐	☐	☐	☐
8	Ayudan a comunicarme con los profesionales.	☐	☐	☐	☐	☐

SUPPLEMENTARY INFORMATION

9	Ayudan a recordarme compartir con el profesional síntomas que se me hubieran olvidado.	☐	☐	☐	☐	☐
10	Ayudan a compartir temas incómodos que me dan vergüenza comunicar normalmente.	☐	☐	☐	☐	☐
11	Ayudan a que los aspectos psicológicos se hablen y se comenten durante la visita.	☐	☐	☐	☐	☐
12	Me tranquilizan.	☐	☐	☐	☐	☐
13	Me provocan angustia.	☐	☐	☐	☐	☐

	**BLOQUE PREMS**<br>En cuanto a los cuestionarios sobre la atención que he recibido que me envía el hospital en mi dispositivo electrónico:	**Totalmente en desacuerdo**	**En desacuerdo**	**Ni de acuerdo ni en desacuerdo**	**De acuerdo**	**Totalmente de acuerdo**
1	Incluyen toda la información relevante para mí.	☐	☐	☐	☐	☐
2	La frecuencia en la que debo responder es adecuada.	☐	☐	☐	☐	☐
3	El tiempo que se tarda en responder es adecuado.	☐	☐	☐	☐	☐
4	Las preguntas son repetitivas.	☐	☐	☐	☐	☐
5	Me gustaría seguir respondiendo cuestionarios sobre la atención que he recibido en el hospital.	☐	☐	☐	☐	☐

	**BLOQUE PRMS**<br>En cuanto a los cuestionarios que me envía el hospital en mi dispositivo electrónico:	**Totalmente en desacuerdo**	**En desacuerdo**	**Ni de acuerdo ni en desacuerdo**	**De acuerdo**	**Totalmente de acuerdo**
1	Considero que la herramienta/programa que se utiliza es fácil de utilizar.	☐	☐	☐	☐	☐
2	La visualización de las preguntas es buena.	☐	☐	☐	☐	☐
3	La redacción y lenguaje de las preguntas es comprensible.	☐	☐	☐	☐	☐
4	Responder preguntas electrónicamente facilita mi participación.	☐	☐	☐	☐	☐

SUPPLEMENTARY INFORMATION

PRM Implementation Assessment Tool – Patients (Catalan)

Qüestionari d'avaluació de la implementació de PROMS i PREMS a la pràctica clínica rutinària a l'Hospital del Mar

Avaluació del programa d'implementació (per a tots els participants)

Instruccions: les següents preguntes fan referència al projecte d'investigació "Implementació de PROMs i PREMs en l'atenció clínica rutinària: avaluació dels seus requisits i impacte" en el qual vostè participa. Les respostes a aquest qüestionari seran tractades de manera anònima. Per contestar, llegeix-hi cada pregunta en l'ordre que apareixen i esculli l'opció de resposta que millor reflecteix la seva opinió.

		Totalment en desacord	En desacord	Ni d'acord, ni en desacord	D'acord	Totalment d'acord
1	Tinc un bon coneixement del meu estat de salut.	☐	☐	☐	☐	☐
2	En les darreres visites, s'ha parlat dels temes importants per el meu dia a dia (ex: problemes de mobilitat, problemes psicològics, altres malalties, impacte de la meva malaltia sobre les meves activitats diàries, pautes d'alimentació i exercici físic, etc.).	☐	☐	☐	☐	☐
3	El/la doctor/a té una visió global (ex: aspectes emocionals, efecte de la malaltia sobre les activitats diàries, etc.) del meu estat de salut relacionat amb la meva malaltia.	☐	☐	☐	☐	☐
4	Em preparo les visites abans d'anar a l'hospital (ex: apuntant-me preguntes o símptomes).	☐	☐	☐	☐	☐
5	La meva participació a l'hospital ha augmentat en els darrers mesos (ex: participació en estudis, en grups de pacients, com a pacient expert, donant la meva opinió, etc.).	☐	☐	☐	☐	☐
6	Estic disposat/ada a respondre qüestionaris sobre el meu estat de salut que m'enviï l'hospital abans de la propera visita.	☐	☐	☐	☐	☐

SUPPLEMENTARY INFORMATION

7	Respondre qüestionaris sobre el meu estat de salut que m'enviï l'hospital millora l'atenció que rebo.	☐	☐	☐	☐	☐
8	Estic disposat/ada a respondre preguntes sobre l'atenció rebuda a l'hospital.	☐	☐	☐	☐	☐
9	Considero útil valorar l'atenció rebuda a l'hospital.	☐	☐	☐	☐	☐
10	Estic disposat/ada a respondre en dispositius electrònics personals (mòbil, ordinador, tablet) a qüestionaris que m'enviï l'hospital.	☐	☐	☐	☐	☐
11	La imatge que tinc de l'hospital ha millorat en els darrers mesos.	☐	☐	☐	☐	☐

Avaluació d'administració

	**BLOC PROMS**<br>Pel que fa als qüestionaris de salut que m'envia l'hospital al meu dispositiu electrònic:	**Totalment en desacord**	**En desacord**	**Ni d'acord, ni en desacord**	**D'acord**	**Totalment d'acord**
1	Inclouen tota la informació rellevant per a mi.	☐	☐	☐	☐	☐
2	La freqüència en la que haig de respondre és adequada.	☐	☐	☐	☐	☐
3	El temps que es tarda a respondre és adequat.	☐	☐	☐	☐	☐
4	Les preguntes són repetitives.	☐	☐	☐	☐	☐
5	Les preguntes s'adeqüen al meu estat de salut actual.	☐	☐	☐	☐	☐
6	Durant la visita, el professional ha comentat amb mi les meves respostes.	☐	☐	☐	☐	☐
7	M'agradaria continuar responent a qüestionaris sobre el meu estat de salut.	☐	☐	☐	☐	☐
8	Ajuden a comunicar-me amb els professionals.	☐	☐	☐	☐	☐
9	Ajuden a enrecordar-me de compartir amb el professional símptomes que se m'haguessin oblidat.	☐	☐	☐	☐	☐

SUPPLEMENTARY INFORMATION

10	Ajuden a compartir temes incomodes que em donen vergonya comunicar normalment.	☐	☐	☐	☐	☐
11	Ajuden a que els aspectes psicològics es parlin i es comentin durant la visita.	☐	☐	☐	☐	☐
12	Em tranquil·litzen.	☐	☐	☐	☐	☐
13	Em provoquen angoixa.	☐	☐	☐	☐	☐

	**BLOC PREMS**<br>Pel que fa als qüestionaris sobre l'atenció que he rebut que m'envia l'hospital al meu dispositiu electrònic:	**Totalment en desacord**	**En desacord**	**Ni d'acord, ni en desacord**	**D'acord**	**Totalment d'acord**
1	Inclouen tota la informació rellevant per a mi.	☐	☐	☐	☐	☐
2	La freqüència en la que haig de respondre és adequada.	☐	☐	☐	☐	☐
3	El temps que es tarda a respondre és adequat.	☐	☐	☐	☐	☐
4	Les preguntes són repetitives.	☐	☐	☐	☐	☐
5	M'agradaria continuar responent a qüestionaris sobre l'atenció que he rebut a l'hospital.	☐	☐	☐	☐	☐

	**BLOC PRMS**<br>Pel que fa als qüestionaris que m'envia l'hospital al meu dispositiu electrònic:	**Totalment en desacord**	**En desacord**	**Ni d'acord, ni en desacord**	**D'acord**	**Totalment d'acord**
1	Considero que l'eina/programa que es fa servir és fàcil d'utilitzar.	☐	☐	☐	☐	☐
2	La visualització de les preguntes és bona.	☐	☐	☐	☐	☐
3	La redacció i llenguatge de les preguntes és comprensible.	☐	☐	☐	☐	☐
4	Respondre preguntes electrònicament facilita la meva participació.	☐	☐	☐	☐	☐

SUPPLEMENTARY INFORMATION

PRM Implementation Assessment Tool – Professionals (English)

<u>Questionnaire to evaluate the implementation of PROMs and PREMs in routine clinical practice at Hospital del Mar</u>

<u>Instructions:</u> The following questions refer to your opinion regarding the research project "Implementation of PROMs and PREMs in routine clinical care: evaluation of its requirements and impact" in which you and your patients participate. Your responses to this questionnaire will be treated anonymously. To answer, read each question in the order they appear and choose the response option that best reflects your opinion.

<u>Definition of PROMs:</u> Any report of an individual's health status that comes directly from that individual, without interpretation of the response by a healthcare professional or any other person.

<u>Definition of PREMs:</u> A measure of an individual's perception of their experience with the healthcare services received.

Age groups:

- ☐ <35
- ☐ 35-44
- ☐ 45-54
- ☐ 55-64
- ☐ 65 and over

Gender:

- ☐ Female
- ☐ Male
- ☐ Other
- ☐ I prefer not to answer

Profession:

- ☐ Doctor
  Specialty:
- ☐ Nurse
- ☐ Case manager
- ☐ Other: ___________

Years of experience in your profession: __________

	**SECTION #1 PRE-IMPLEMENTATION**	**Strongly disagree**	**Disagree**	**Neither agree nor disagree**	**Agree**	**Strongly agree**
1	I understand what a PR**O**M is	☐	☐	☐	☐	☐
2	I can interpret the results of PR**O**Ms	☐	☐	☐	☐	☐

SUPPLEMENTARY INFORMATION

3	I am willing to implement PR**O**Ms in my routine clinical practice	☐	☐	☐	☐	☐
4	In case you are not willing, why not? Answer options: lack of time, I don't believe they are useful, I don't want to change my work methodology, others (open field)	☐	☐	☐	☐	☐
5	I understand what a PR**E**M is	☐	☐	☐	☐	☐

**SECTION #2 PRE AND POST-IMPLEMENTATION** Regarding the implementation of PROMs and PREMs in clinical practice...		**Strongly disagree**	**Disagree**	**Neither agree nor disagree**	**Agree**	**Strongly agree**
6	My patients are actively involved in managing their illness (attending all visits, adhering to treatment, making decisions, etc.).	☐	☐	☐	☐	☐
7	At the end of the visit, I get an overall view of the patient's health status related to their illness.	☐	☐	☐	☐	☐
8	In the visit, I identify the main need/priority of the patient related to their illness	☐	☐	☐	☐	☐
9	I am satisfied with the quality of my care towards patients	☐	☐	☐	☐	☐
10	I conduct a standardized visit for patients who are in a similar stage of their illness.	☐	☐	☐	☐	☐
11	The communication with my patients is fluid.	☐	☐	☐	☐	☐
12	The PR**O**Ms used collect all relevant information from the patient.	☐	☐	☐	☐	☐
13	Currently, I use the results of PR**O**Ms in my routine clinical practice.	☐	☐	☐	☐	☐
14	The implementation of PR**O**Ms provides a benefit for patients.	☐	☐	☐	☐	☐
15	The implementation of PR**O**Ms provides a benefit for professionals.	☐	☐	☐	☐	☐
16	The implementation of PR**O**Ms provides a benefit for the institution/department/service.	☐	☐	☐	☐	☐
17	I have sufficient training for the interpretation and use of PR**O**Ms.	☐	☐	☐	☐	☐

SUPPLEMENTARY INFORMATION

18	My institution is prepared to incorporate PR**O**Ms into clinical practice.	☐	☐	☐	☐	☐
19	The implementation of PR**E**Ms provides a benefit for patients.	☐	☐	☐	☐	☐
20	The implementation of PR**E**Ms provides a benefit for professionals.	☐	☐	☐	☐	☐
21	The implementation of PR**E**Ms provides a benefit for the institution/department/service.	☐	☐	☐	☐	☐
22	The use of PR**O**Ms will be incorporated into routine clinical practice	☐	☐	☐	☐	☐
23	The use of PR**E**Ms will be incorporated into routine clinical practice	☐	☐	☐	☐	☐

**SECTION #3 POST-IMPLEMENTACIÓN**		**Strongly disagree**	**Disagree**	**Neither agree nor disagree**	**Agree**	**Strongly agree**
24	The incorporation of PR**O**Ms has improved my overall view of the patient	☐	☐	☐	☐	☐
25	With the use of PR**O**Ms, I can detect problems and symptoms sooner	☐	☐	☐	☐	☐
26	I have incorporated the use of PR**O**Ms into my regular clinical practice	☐	☐	☐	☐	☐
27	After the study, the institution should support the implementation of PR**O**Ms and PR**E**Ms	☐	☐	☐	☐	☐
28	I will continue using PR**O**Ms and PR**E**Ms beyond the study	☐	☐	☐	☐	☐
29	I have known whom to contact for questions regarding the implementation program of PR**O**Ms	☐	☐	☐	☐	☐
30	I have had sufficient support from the research team when needed	☐	☐	☐	☐	☐
31	The PR**O**Ms administration' frequency is adequate	☐	☐	☐	☐	☐
32	The PR**O**Ms' results are easily accessible	☐	☐	☐	☐	☐
33	The PR**O**Ms'results are easily interpretable.	☐	☐	☐	☐	☐
34	*Specific to each implementation program*					

OPEN QUESTIONS

- The advantages of the PR**O**Ms and PR**E**Ms implementation program are:

SUPPLEMENTARY INFORMATION

- The disadvantages of the PR**O**Ms and PR**E**Ms implementation program are:

☐ Lack of time
☐ Access to PROMs results
☐ Training for the interpretation of PROMs
☐ Others:

- How could the usability of the PR**O**Ms system be improved?

- What have you missed in the PR**O**Ms computer system?

SUPPLEMENTARY INFORMATION

PRM Implementation Assessment Tool – Professionals (Spanish)

Cuestionario de evaluación de la implementación de PROMS y PREMS a la práctica clínica rutinaria en el Hospital del Mar

Instrucciones: Las siguientes preguntas se refieren a su opinión sobre el proyecto de investigación "Implementación de PROMs y PREMs en la atención clínica rutinaria: evaluación de sus requisitos e impacto" en el cual usted y sus pacientes participan. Las respuestas a este cuestionario serán tratadas de manera anónima. Para responder, lea cada pregunta en el orden que aparecen y elija la opción de respuesta que mejor refleje su opinión.

Definición de PROMs: cualquier informe del estado de salud de un individuo, que proviene directamente de ese individuo, sin interpretación de la respuesta por parte de un profesional o cualquier otra persona.

Definición de PREMs: una medida de la percepción del individuo sobre su experiencia con la atención recibida en los servicios sanitarios.

SUPPLEMENTARY INFORMATION

Grupo de edad:
- ☐ <35
- ☐ 35-44
- ☐ 45-54
- ☐ 55-64
- ☐ 65 o más

Genero:
- ☐ Femenino
- ☐ Masculino
- ☐ Otro
- ☐ Prefiero no contestar

Grupo profesional:
- ☐ Médico/a
  Especialidad: ________
- ☐ Enfermero/a
- ☐ Gestor/a de casos
- ☐ Otro
  Cuál: ___________

Años de experiencia en su profesión: __________

	**BLOC 1 PRE-IMPLEMENTACIÓN**	**Totalmente en desacuerdo**	**En desacuerdo**	**Ni de acuerdo, ni en desacuerdo**	**De acuerdo**	**Totalmente de acuerdo**
1	Entiendo lo que es un PR**O**M	☐	☐	☐	☐	☐
2	Sé interpretar los resultados de los PR**O**Ms.	☐	☐	☐	☐	☐

3	Estoy dispuesto/a a implementar los PR**O**Ms en mi práctica clínica habitual	☐	☐	☐	☐	☐
4	En caso de que no esté dispuesto/a, ¿por qué no?<br>Opciones de respuesta: NA, falta de tiempo, no creo que sean útiles, no quiero cambiar mi metodología de trabajo, otros (campo abierto)	☐	☐	☐	☐	☐
5	Entiendo lo que es un PR**E**M	☐	☐	☐	☐	☐

**BLOC 2 PRE Y POST-IMPLEMENTACIÓN Sobre la implementación de PROMs y PREMs en la práctica clínica...**		**Totalmente en desacuerdo**	**En desacuerdo**	**Ni de acuerdo, ni en desacuerdo**	**De acuerdo**	**Totalmente de acuerdo**
6	Mis pacientes están implicados de manera activa en el manejo de su enfermedad (asisten a todas las visitas, siguen el tratamiento y sugieren cambios, toman decisiones, etc.)	☐	☐	☐	☐	☐
7	Al finalizar la visita, obtengo una visión global del estado de salud del/de la paciente relacionado con su enfermedad	☐	☐	☐	☐	☐
8	En la visita identifico la principal necesidad/prioridad del/de la paciente relacionada con su enfermedad	☐	☐	☐	☐	☐
9	Estoy satisfecho/a con la calidad de mi atención hacia los pacientes	☐	☐	☐	☐	☐
10	Hago una visita estandarizada en los/las pacientes que están en una etapa similar de su enfermedad	☐	☐	☐	☐	☐
11	La comunicación con mis pacientes es fluida	☐	☐	☐	☐	☐
12	Los PR**O**Ms utilizados recogen toda la información relevante por parte del/de la paciente	☐	☐	☐	☐	☐
13	Actualmente uso los resultados de PR**O**Ms en la práctica clínica habitual	☐	☐	☐	☐	☐

SUPPLEMENTARY INFORMATION

14	La implementación de PR**O**Ms supone un beneficio para los/las pacientes	☐	☐	☐	☐	☐
15	La implementación de PR**O**Ms supone un beneficio para los/las profesionales.	☐	☐	☐	☐	☐
16	La implementación de PR**O**Ms supone un beneficio para la institución/departamento/servicio.	☐	☐	☐	☐	☐
17	Tengo suficiente formación para la interpretación y uso de PR**O**Ms	☐	☐	☐	☐	☐
18	Mi institución está preparada para incorporar PR**O**Ms a la práctica clínica	☐	☐	☐	☐	☐
19	La implementación de PR**E**Ms supone un beneficio para los/ las pacientes	☐	☐	☐	☐	☐
20	La implementación de PR**E**Ms supone un beneficio para los/ las profesionales	☐	☐	☐	☐	☐
21	La implementación de PR**E**Ms supone un beneficio para la institución/departamento/servicio	☐	☐	☐	☐	☐
22	El uso de PR**O**Ms se incorporará en la práctica clínica habitual.	☐	☐	☐	☐	☐
23	El uso de PR**E**Ms se incorporará en la práctica clínica habitual.	☐	☐	☐	☐	☐

**BLOC 3 PREGUNTAS POST-INTERVENCIÓN**		**Totalmente en desacuerdo**	**En desacuerdo**	**Ni de acuerdo, ni en desacuerdo**	**De acuerdo**	**Totalmente de acuerdo**
24	La incorporación de los PR**O**Ms ha mejorado mi visión global del paciente	☐	☐	☐	☐	☐
25	Con el uso de PR**O**Ms puedo detectar problemas y síntomas más temprano	☐	☐	☐	☐	☐
26	He incorporado el uso de PR**O**Ms en mi práctica clínica habitual	☐	☐	☐	☐	☐
27	Después del estudio, la institución debería apoyar la implementación de PR**O**Ms y PR**E**Ms	☐	☐	☐	☐	☐
28	Continuaré utilizando PR**O**Ms y PR**E**Ms más allá del estudio	☐	☐	☐	☐	☐

SUPPLEMENTARY INFORMATION

29	He sabido a quién dirigirme para hacer las preguntas que tengo sobre el funcionamiento del programa de implementación de PR**O**Ms	☐	☐	☐	☐	☐
30	He tenido suficiente apoyo del equipo investigador cuando lo necesito	☐	☐	☐	☐	☐
31	La frecuencia de administración de los PR**O**Ms es adecuada	☐	☐	☐	☐	☐
32	Los resultados de los PR**O**Ms son fácilmente accesibles	☐	☐	☐	☐	☐
33	Los resultados de los PR**O**Ms son fácilmente interpretables	☐	☐	☐	☐	☐
34	*Específico para cada programa de implementación*	☐	☐	☐	☐	☐

PREGUNTAS ABIERTAS

- Las ventajas del programa de implementación de PROMs y PREMs son:

- Los inconvenientes del programa de implementación de PROMs y PREMs son:

☐ Falta de tiempo
☐ Acceso a los resultados de PROMs
☐ Formación para la interpretación de PROMs
☐ Otros:

- ¿Cómo se podría mejorar la usabilidad del sistema de PROMs?

- ¿Qué has hechado de menos en el sistema informático de PROMs?

SUPPLEMENTARY INFORMATION

PRM Implementation Assessment Tool – Professionals (Catalan)

Qüestionari d’avaluació de la implementació de PROMS i PREMS a la pràctica clínica rutinària a l’Hospital del Mar

Instruccions: Les següents preguntes fan referència a la seva opinió respecte el projecte d’investigació “Implementació de PROMs i PREMs en l’atenció clínica rutinària: avaluació dels seus requisits i impacte” en el qual vostè i els seus / les seves pacients participen. Les respostes a aquest qüestionari seran tractades de manera anònima. Per contestar, llegeix-hi cada pregunta en l’ordre que apareixen i esculli l’opció de resposta que millor reflecteix la seva opinió.

Definició de PROMs: qualsevol informe de l’estat de salut d’un individu, que prové directament d’aquest individu, sense interpretació de la resposta per part d’un professional o qualsevol altra persona.
Definició de PREMs: una mesura de la percepció de l’individu sobre la seva experiència amb l’atenció rebuda als serveis sanitaris.

Grup d’edat:

- ☐ <35
- ☐ 35-44
- ☐ 45-54
- ☐ 55-64
- ☐ 65 i més

Gènere:

- ☐ Femení
- ☐ Masculí
- ☐ Altre
- ☐ Prefereixo no contestar

Grup professional:

- ☐ Metge/ssa
  Especialitat:
- ☐ Infermer/era
- ☐ Gestor/a de casos
- ☐ Altre: ___________

Anys d’experiència en la seva professió: __________

	BLOC 1 PRE-IMPLEMENTACIÓ	Totalment en desacord	En desacord	Ni d’acord, ni en desacord	D’acord	Totalment d’acord
1	Entenc el que és un PR**O**M	☐	☐	☐	☐	☐
2	Sé interpretar els resultats dels PR**O**Ms	☐	☐	☐	☐	☐
3	Estic disposat/ada a implementar els PR**O**Ms a la meva pràctica clínica habitual	☐	☐	☐	☐	☐

SUPPLEMENTARY INFORMATION

4	En cas de que no estigui disposat/ada, perquè no?<br>Opcions de resposta: falta de temps, no crec que siguin útils, no vull canviar la meva metodologia de treball, altres (camp obert)	☐	☐	☐	☐	☐
5	Entenc el que és un PR**E**M	☐	☐	☐	☐	☐

**BLOC 2 PRE I POST-IMPLEMENTACIÓ**<br>Sobre la implementació de PROMs i PREMs en la pràctica clínica...		**Totalment en desacord**	**En desacord**	**Ni d'acord, ni en desacord**	**D'acord**	**Totalment d'acord**
6	Els meus pacients estan implicats de manera activa en el maneig de la seva malaltia (venir a totes les visites, adherència i suggeriments al tractament, presa de decisions , ...)	☐	☐	☐	☐	☐
7	A l'acabar la visita aconsegueixo una visió global de l'estat de salut del/ de la pacient relacionat amb la seva malaltia	☐	☐	☐	☐	☐
8	A la visita identifico la principal necessitat/prioritat del/ de la pacient relacionada amb la seva malaltia	☐	☐	☐	☐	☐
9	Estic satisfet/a amb la qualitat de la meva atenció envers als pacients	☐	☐	☐	☐	☐
10	Faig una visita estandarditzada en els/les pacients que estan en un estadi similar de la seva malaltia.	☐	☐	☐	☐	☐
11	La comunicació amb els meus/ les meves pacients és fluida	☐	☐	☐	☐	☐
12	Els PR**O**Ms utilitzats recullen tota la informació rellevant per part del/ de la pacient	☐	☐	☐	☐	☐
13	Actualment faig servir els resultats de PR**O**Ms en la pràctica clínica habitual	☐	☐	☐	☐	☐
14	La implementació de PR**O**Ms suposa un benefici per als/ a les pacients	☐	☐	☐	☐	☐
15	La implementació de PR**O**Ms suposa un benefici per als/ a les professionals	☐	☐	☐	☐	☐
16	La implementació de PR**O**Ms suposa un benefici per a la institució/departament/servei	☐	☐	☐	☐	☐
17	Tinc suficientment formació per a la interpretació i ús de PR**O**Ms	☐	☐	☐	☐	☐
18	La meva institució està preparada per incorporar PR**O**Ms a la pràctica clínica	☐	☐	☐	☐	☐

SUPPLEMENTARY INFORMATION

19	La implementació de PR**E**Ms suposa un benefici per als/ a les pacients	☐	☐	☐	☐	☐
20	La implementació de PR**E**Ms suposa un benefici per als/ a les professionals	☐	☐	☐	☐	☐
21	La implementació de PR**E**Ms suposa un benefici per a la institució/departament/servei	☐	☐	☐	☐	☐
22	L'ús de PR**O**Ms s'incorporarà a la pràctica clínica habitual	☐	☐	☐	☐	☐
23	L'ús de PR**E**Ms s'incorporarà a la pràctica clínica habitual	☐	☐	☐	☐	☐

**BLOC 3 POST-IMPLEMENTACIÓ**		**Totalment en desacord**	**En desacord**	**Ni d'acord, ni en desacord**	**D'acord**	**Totalment d'acord**
24	La incorporació dels PR**O**Ms ha millorat la meva visió global del pacient	☐	☐	☐	☐	☐
25	Amb l'ús de PR**O**Ms puc detectar problemes i símptomes més aviat	☐	☐	☐	☐	☐
26	He incorporat l'ús de PR**O**Ms en la meva pràctica clínica habitual	☐	☐	☐	☐	☐
27	Després de l'estudi, la institució hauria de donar suport a la implementació de PROMs i PREMs	☐	☐	☐	☐	☐
28	Continuaré utilitzant PROMs i PREMs més enllà de l'estudi	☐	☐	☐	☐	☐
29	He sapigut a qui m'he de dirigir per fer les preguntes que tinc sobre el funcionament del programa d'implementació de PR**O**Ms	☐	☐	☐	☐	☐
30	He tingut suficient suport de l'equip investigador quan el necessito	☐	☐	☐	☐	☐
31	La freqüència d'administració dels PR**O**Ms és adequada	☐	☐	☐	☐	☐
32	Els resultats dels PR**O**Ms són fàcilment accessibles	☐	☐	☐	☐	☐
33	Els resultats dels PR**O**Ms són fàcilment interpretables	☐	☐	☐	☐	☐
34	*Específic de cada programa d'implementació*					

PREGUNTES OBERTES

- Les avantatges del programa d'implementació de PROMs i PREMs són:

- Els inconvenients del programa d'implementació de PROMs i PREMs són:

SUPPLEMENTARY INFORMATION

☐ Falta de temps
☐ Accés als resultats de PROMs
☐ Formació per a la interpretació de PROMs
☐ Altres:

- Com es podria millorar la usabilitat del sistema de PROMs?

- Què has trobat a faltar en el sistema informàtic de PROMs?

SUPPLEMENTARY INFORMATION
